# The association between obesity with serum levels of liver enzymes, alanine aminotransferase, aspartate aminotransferase, alkaline phosphatase and gamma‐glutamyl transferase in adult women

**DOI:** 10.1002/edm2.367

**Published:** 2022-08-30

**Authors:** Vahideh Jalili, Zohreh Poorahmadi, Naeemeh Hasanpour Ardekanizadeh, Maryam Gholamalizadeh, Marjan Ajami, Anahita Houshiarrad, Azadeh Hajipour, Fatemeh Shafie, Atiyeh Alizadeh, Zohreh Mokhtari, Hanieh Shafaei, Mina Esmaeili, Saeid Doaei

**Affiliations:** ^1^ Faculty of Medicine Urmia University of Medical sciences Urmia Iran; ^2^ Department of Food and Beverage Products Food and Drug Administration Tehran Iran; ^3^ Department of Clinical Nutrition, School of Nutrition and Food Sciences Shiraz University of Medical Sciences Shiraz Iran; ^4^ Cancer Research Center Shahid Beheshti University of Medical Sciences Tehran Iran; ^5^ Department of Food and Nutrition Policy and Planning National Nutrition and Food Technology Research Institute, School of Nutrition Sciences and Food Technology, Shahid Beheshti University of Medical Sciences Tehran Iran; ^6^ Department of Nutrition Research National Nutrition and Food Technology Research Institute, School of Nutrition Sciences and Food Technology, Shahid Beheshti University of Medical Science Tehran Iran; ^7^ School of Health Qazvin University of Medical Sciences Qazvin Iran; ^8^ Nutrition Research Center, School of Nutrition and Food Sciences Shiraz University of Medical Sciences Shiraz Iran; ^9^ Department of Pharmacologynosy, Faculty of Pharmacy Tehran University of Medical Sciences Tehran Iran; ^10^ Department of Clinical Biochemistry, Faculty of Medicine Shahid Beheshti University of Medical Sciences Tehran Iran; ^11^ Nursing and Midwifery School Guilan University of Medical Sciences Rasht Iran; ^12^ Department of Nutrition Research National Nutrition and Food Technology Research Institute, School of Nutrition Sciences and Food Technology, Shahid Beheshti University of Medical Sciences Tehran Iran; ^13^ Department of Community Nutrition National Nutrition and Food Technology Research Institute, Faculty of Nutrition Sciences and Food Technology, Shahid Beheshti University of Medical Sciences Tehran Iran

**Keywords:** body mass index, liver enzyme, liver function, obesity

## Abstract

**Background:**

Obesity‐induced inflammation may independently disturb the function of critical organs such as liver. This study aimed to investigate the association of obesity with serum levels of biomarkers of liver function including alanine aminotransferase (ALT), aspartate aminotransferase (AST), alkaline phosphatase (ALP) and gamma‐glutamyl transferase (GGT) in adult women.

**Methods:**

This cross‐sectional study was carried out on 360 adult women in the summer of 2020 in Tehran, Iran. The participants were categorized into two groups based on their body mass index (BMI≤29.9 and BMI > 30). The serum levels of ALT, AST, ALP and GGT were measured. Logistic regression method was used to assess the association between BMI and liver enzymes after adjusting for the confounders.

**Results:**

The mean BMI in non‐obese and obese groups was 26.32 ± 2.61 and 33.40 ± 2.80 kg/m^2^, respectively (*p* = .01). A significant association was found between BMI with ALT (*β* = .16, *p* = .002) and GGT (*β* = .19, *p* = .01) enzymes after adjustment for age. The association between BMI and GGT remained significant after further adjustments for smoking, alcohol use, physical activity and educational status. There was no significant association between BMI and liver enzymes after adjustment for dietary intake.

**Conclusions:**

Obesity was associated with the level of serum liver enzymes. However, adjustment for dietary intake disappeared the significant results. Further studies are needed to determine the independent effects of obesity on the liver function.

## Graphical abstract

The results of the present study indicated that there was a positive association between BMI and serum level of GGT and ALT after adjustment with confounding variable of age. The association between BMI and GGT remained significant after adjusting for smoking status, alcohol use, physical activity, and educational status. There was no significant association between BMI and liver enzymes after further adjustment for dietary intake of calorie, protein, carbohydrate, and fat.

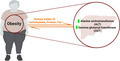

## 1 INTRODUCTION

The dramatic increase in obesity remained challenging worldwide, and it has been estimated that about 40% of world population will be overweight and 20% will be obese by 2030 ^1^ ^2^. Obesity is a well‐known risk factor for metabolic syndrome which may lead to chronic diseases such as diabetes, cardiovascular diseases and non‐alcoholic fatty liver disease (NAFLD).^3^, ^4^ Recent studies reported that obesity may be associated with liver disease and progression of hepatic dysfunction, and obesity may impair liver function by a variety of mechanisms. In individuals with obesity, high levels of cytokines including interleukin‐6 (IL‐6) and C‐reactive protein (CRP) may disrupt liver functions such as production of hepcidin which can lead to hepcidin‐related iron deficiency anaemia^5^, ^6^, ^7^, ^8^ and may lead to some types of liver diseases such as NAFLD and liver cancer.^6^, ^9^, ^10^, ^11^, ^12^

The serum levels of four enzymes including alanine aminotransferase (ALT), aspartate aminotransferase (AST), alkaline phosphatase (ALP) and γ‐glutamyl transferase (GGT) are generally used in assessing liver functions.^13^ ALT and AST are found mostly in the liver, and serum levels of AST and ALT are considered as specific markers for hepatic dysfunction.^14^ GGT is present in the cell membranes of many tissues, with greatest activity in biliary epithelial cells, pancreatic acinar cells and renal tubular epithelial cells. ALP is an enzyme that is primarily present in the liver, bones, intestine and kidneys^15^

Previously, some studies have been carried out to evaluate the relationship of ALT and GGT with obesity.^16^, ^17^, ^18^ A positive correlation was reported for abdominal obesity with ALT and GGT in previous studies.^19^, ^20^ Serum levels of ALT showed a significant association with only general obesity in the regression models, whereas GGT showed a significant relationship with both general and abdominal obesity. In all models of the regression analysis, serum GGT showed a stronger association with obesity than the other liver enzymes^21^

The higher serum levels of ALT, AST, ALP and GGT are reported in several diseases and increased levels of these enzymes are frequently reported in people with obesity.^22^, ^23^ Interestingly, the results of a recent meta‐analysis reported that serum activity of liver enzymes is associated with higher mortality in coronavirus disease (COVID‐19). Another study found that obesity causes metabolic disorders such as high fasting blood glucose and insulin resistance through increasing levels of ALT and GGT enzymes, and this association is more pronounced in women than men.^24^ The association between obesity and serum biomarkers of liver function, independent of dietary intake and physical activity, is not yet clear. Due to the role of liver enzymes in the body's metabolism,^25^, ^26^, ^27^ if the link between obesity and serum levels of liver enzymes is proven, this finding may be a clue as a possible mechanism by which obesity plays a role in the risk of a broad range of diseases such as fatty liver, diabetes and cancers. So, this study aimed to investigate the association of obesity with serum levels of AST, ALT, ALP and GGT in Iranian adult women. The hypothesis of this study was that weight gain is independently associated with the increased levels of liver enzymes in adult women.

## 2 METHODS

This cross‐sectional study was carried out on 360 healthy adult women from September 2020 to March 2021 in Tehran, Iran. The samples were randomly selected through informing on social networks. Inclusion criteria were the age of 35–65 years and consent to participate in the study. Exclusion criteria were no access of blood sample and/or laboratory results, suffering from diseases affecting weight, taking drugs affecting weight, and lack of sufficient information to calculate BMI. Data related to demographic and social indices were collected via general questionnaire. Height and weight of individuals were measured by stadiometer and Seca scale, respectively, and BMI was calculated as weight (in kilograms) divided by height (in meters) squared. Amount of physical activity was assessed by international physical activity questionnaire.^28^ Also, the intakes of calorie, protein, carbohydrate and fat from were assessed by a validated food frequency questionnaire.^29^

### 2.1 Laboratory assessment

Five millilitres of blood samples were collected from the participants in the fasted state. Serum levels of GGT, ALP, ALT and AST were determined using an auto‐analyser (BT1500; Biotecnica Instrument,) and Pars Azmun standard kits.

### 2.2 Statistical analysis

All participants were categorized into two groups of obese (BMI > 30) and non‐obese (BMI≤29.9). Continuous and categorical variables were compared between the groups using independent t‐test and chi‐square tests, respectively. Linear regression was applied in different models to examine the linear association between BMI and serum liver enzyme levels. Age was adjusted in the first model. Further adjustments for smoking (yes/no), alcohol use (yes/no), physical activity (sedentary/moderate/active) and education (uneducated/elementary/junior high school/high school diploma/university or college degree/postgraduate) were performed in the second model. Additional adjustment for dietary intake was performed in the third model. All statistical analyses were done using the Statistical Package for Social Science version 21 (SPSS Inc, Chicago), and *p* < .05 was considered statistically significant.

## 3 RESULTS

General characteristics of the participants are presented in Table 1. The mean BMI in non‐obese and obese groups were 26.32 ± 2.61 and 33.40 ± 2.80 kg/m^2^, respectively (*p* = .01). No significant difference was found in terms of age, height, physical activity and dietary intake of calorie, protein, carbohydrate and fat.

**TABLE 1:** General characteristics of the participants

	Normal weight (*n* = 213)	Obese (*n* = 140)	*p*
Age (year)	50.33 ± 9.33	51.32 ± 8.56	.27
Height (m)	157.38 ± 6.18	156.30 ± 5.47	.06
Weight (Kg)	65.26 ± 8.00	81.68 ± 8.68	.01
BMI (kg/m^2^)	26.32 ± 2.61	33.40 ± 2.80	.01
Physical activity (hour)	1.54 ± 1.53	1.55 ± 2.74	.96
Calorie intake (Kcal)	2591.51 ± 1036.91	2480.99 ± 937.75	.53
Protein intake (g)	86.90 ± 44.08	83.01 ± 34.28	.56
Carbohydrate intake (g)	366.37 ± 152.63	355.98 ± 148.84	.70
Fat intake (g)	95.89 ± 50.40	87.69 ± 38.31	.28

The comparison of levels of serum liver enzymes is presented in Table 2. The people with obesity had higher levels of ALT (17.17 ± 7.54 vs. 19.55 ± 9.79 IU/L, *p* = .02), ALP (217.0 ± 68.34 vs. 237.113 ± 72.42 IU/L, *p* = .01) and GGT (18.82 ± 12.94 vs. 21.78 ± 13.24 IU/L, *p* = .04) compared with the non‐obese people (Table 2). No significant difference was found on the serum level of AST between two groups.

**TABLE 2:** Levels of liver enzymes between normal and overweight participants

	Normal weight (*n* = 213)	Obese (*n* = 140)	*p*
AST (IU/L)	18.59 (±4.98)	18.83 (±5.31)	.67
ALT (IU/L)	17.17 (±7.54)	19.55 (±9.79)	.02
ALP (IU/L)	217.077 (±68.34)	237.113 (±72.42)	.01
GGT (IU/L)	18.82 (±12.94)	21.78 (±13.24)	.04

Abbreviations: AST, serum glutamate‐oxaloacetate transferase; ALT, serum glutamate‐pyruvate transferase; ALP, alkaline phosphatase; GGT, gamma‐glutamyl transferase; IU/L, international units per litre.

**FIGURE 1:**
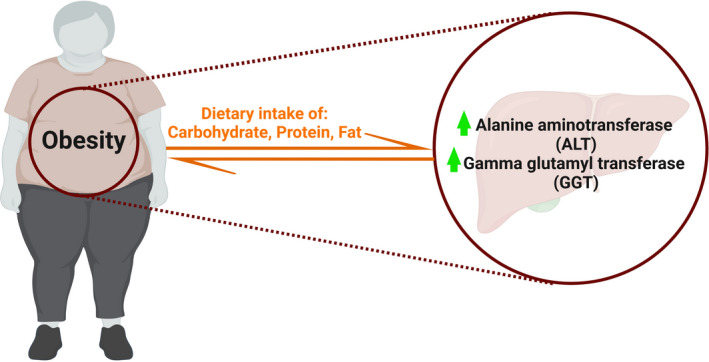
The association between liver enzyme and obesity

Logistic regression method identified a significant positive association between BMI and ALT (*β* = .16, *p* = .002) and GGT (*β* = .19, *p* = .01) enzymes after adjustment for age (Table 3). The association between BMI and GGT remained significant after further adjustments for smoking status, alcohol use, physical activity and educational status (Model 2). There was no significant association between BMI and liver enzymes after adjustment for dietary intake of calorie, protein, carbohydrate and fat (Model 3). The obtained results showed that after adjustment of dietary intake, there was no significant relationship between BMI and serum level of liver enzymes Figure 1.

**TABLE 3:** Linear regression of the association between BMI and liver enzymes

	AST		ALT		ALP		GGT	
	B	*p*	B	*p*	B	*p*	B	*p*
Model 1	0.16	.76	0.16	.002	0.06	.19	0.19	.01
Model 2	−0.01	.92	0.14	.01	0.05	.31	0.18	.001
Model 3	−0.12	.92	0.15	.18	0.03	.72	−0.29	.80

*Note*: Model 1: adjusted for BMI and age, Model 2: adjusted for smoking status, alcohol use, physical activity, and educational status, Model 3: adjusted for intake of calorie, protein, carbohydrate, and fat.

## 4 DISCUSSION

The results of the present study indicated that there was a positive association between BMI and serum level of GGT and ALT after adjustment with confounding variables of age, smoking status, alcohol use, physical activity and educational status. There was no significant association between BMI and liver enzymes after further adjustment for dietary intake of calorie, protein, carbohydrate and fat. Therefore, the relationship between obesity and the level of liver enzymes may be related to the effect of macronutrient intake on liver function.

In line with our study, several studies reported that obesity may be associated with liver diseases such as non‐alcoholic liver diseases (NAFLD).^30^ Duseja et al. in a study on 1168 Indian people found that NAFLD was higher in people with BMI higher than 25 kg/m2.^31^ Marchesini et al. indicated that the increased levels of hepatic enzymes including ALT, AST and GGT are more common in people with obesity.^32^ Another study identified that the subjects with obesity had significantly higher levels of GGT and ALP and reported that GGT had the strongest association with BMI.^33^ Nurshad et al. reported that the mean level of serum ALT, AST and GGT was significantly higher in the group with obesity than the group with normal BMI. Serum GGT showed a significant association with both general and abdominal obesity.^34^ Das et al. found that ALT, AST and GGT levels were higher in individuals with obesity, but no direct association was found between these liver enzymes with obesity.^35^

The underlying mechanisms of the possible association between obesity and the serum level of liver enzymes are not yet clear. Elevated liver enzymes in women with overweight and obesity compared with the normal‐weight women can be associated with weight‐related hormonal disorders such as polycystic ovary syndrome and higher levels of free androgen and total testosterone which are prevalent in women with obesity.^36^ The increased serum level of aminotransferases and especially ALT was frequently reported in PCOS women.^37^ Interestingly, Xu et al. reported that the effect of obesity on diabetes is partly mediated by GGT and ALT but not AST.^38^ Another study found that obesity may increase DNA methylation in liver tissue by increasing oxidative stress and ultimately lead to liver tissue destruction.^39^ Moreover, visceral adipose tissues secrete a variety of proteins such as adipokines, resistin, leptin, visfatin and tumour necrosis factor α which can influence the liver function and lead to inflammation, cirrhosis and hepatocellular cancer.^40^ However, there are also several factors that may mutually affect obesity and liver function such as dietary intake.^41^, ^42^, ^43^ The results of the present study indicated that there was no significant association between BMI and liver enzymes after adjustment for the amount of calorie and macronutrient intake. This finding suggests that macronutrient and calorie intake rather than obesity may be the main cause of impaired liver enzyme serum levels.

However, this study had some limitations. This study was cross‐sectional, and the participants were limited to women. In addition, other biochemical and histological indicators of liver function were not evaluated. Further longitudinal studies on both genders using different liver function biomarkers are needed to determine the effects of obesity on liver function in adults and to discover the underlying mechanisms.

## 5 CONCLUSIONS

These results indicated an association between BMI with ALT and GGT after adjustment with confounding variables of age, smoking status, alcohol use, physical activity and educational status. However, there was no significant association between BMI and liver enzymes after further adjustment for dietary intake. It is plausible that macronutrient and calorie intake rather than obesity may be the main cause of impaired liver enzyme serum levels. Further studies are needed to determine the independent effects of obesity on liver function.

## AUTHOR CONTRIBUTIONS

**Vahideh Jalili:** Formal analysis (equal). **Zohreh Poorahmadi:** Formal analysis (equal). **Naeemeh Hasanpour Ardekanizadeh:** Validation (equal). **Marjan Ajami:** Validation (equal). **Anahita Houshiarrad:** Writing – original draft (equal). **Azadeh Hajipour:** Supervision (equal). **Atiyeh Alizadeh:** Investigation (equal). **Zohreh Mokhtari:** Formal analysis (equal). **Hanieh Shafaei:** Validation (equal). **Mina Esmaeili:** Data curation (equal).

## FUNDING INFORMATION

Funding for this study was provided by National Nutrition and Food Technology Research Institute, Faculty of Nutrition Sciences and Food Technology, Shahid Beheshti University of Medical Sciences, Tehran, Iran.

## CONFLICT OF INTEREST

The authors declare that they have no competing interests.

## ETHICAL APPROVAL AND CONSENT TO PARTICIPATE

All patients signed an informed consent form at baseline. This study was approved by the ethical committee of Shahid Beheshti University of Medical Sciences, Tehran, Iran (Code: IR.SBMU.NNFTRI.REC.1401.028(.

## CONSENT FOR PUBLICATION

Institutional consent forms were used in this study.

## Data Availability

The datasets used during the current study are available from the corresponding author on reasonable request.
